# Aortic valve calcification: a bone of contention

**DOI:** 10.1093/eurheartj/ehw071

**Published:** 2016-03-18

**Authors:** Sergio Bertazzo, Eileen Gentleman

**Affiliations:** 1 Department of Medical Physics & Biomedical Engineering, University College London, Malet Place Engineering Building, London WC1E 6BT, UK; 2 Craniofacial Development and Stem Cell Biology, King's College London, London SE1 9RT, UK

## Introduction

Pathologists have known for centuries that calcific deposits formed in vascular tissues such as the aortic valve. Calcific deposits are hard like bone and have often been described as such. In his ‘History of Animals’, Aristotle remarked that often in oxen and particularly in horse, the heart ‘has a bone inside it’.^1^ And in 1646, the French professor of medicine Lazare Riviére described his post-mortem examination of a patient's diseased aortic valve by remarking that it resembled ‘a cluster of hazelnuts’.^2^ Today, aortic valve calcification remains the most common cause of valve stenosis^3,4^ and is present in some 26% of the population over the age of 65, and as many as half of those over 85.^5^

Well into the 20th century, the formation of calcific lesions was believed to be a normal, passive process associated with aging. In the early 1990s, however, this attitude began to change when Boström *et al*. identified bone morphogenetic protein-2a in calcified human carotid arteries.^6^ Later, in addition to descriptions of standard ‘dystrophic calcification’, histological studies identified endochondral bone formation in heavily calcified aortic valves^7^ and gene expression analyses demonstrated up-regulation of several bone-specific genes.^8^ The biological case for bone formation in the vascular system had been established, but how closely do calcific lesions resemble bone as a material?

To understand bone, biomineralization researchers have long utilized non-quantitative physical science techniques, including analytical electron microscopy, to examine tissues *ex vivo*. Such analyses can sometimes reveal more than standard histological stains that often only detect the presence of calcium and/or phosphate. In particular, standard histology does not definitively identify the specific type of mineral present, as calcium phosphate can exist in >10 different phases.^9^ The mollusc-smashing club that forms part of the exoskeleton of the variegated peacock mantis shrimp, for example, is partially formed of calcium phosphate,^10^ but fundamentally differs from bone. By applying such characterization techniques to bone, we know a great deal about its structure, composition, and mechanism of formation. However, only recently have similar techniques been applied to calcific lesions on the aortic valve. Here, we examine this new evidence and compare it with similar studies conducted on bone. These analyses lead us to believe that ubiquitous features of calcific lesions cannot be explained either by classic descriptions of dystrophic calcification or simply by ectopic bone formation. Consequently, we advocate complementing current biologically focussed research endeavours with interdisciplinary approaches to help disentangle the interwoven biological and physicochemical processes that drive aortic valve calcification with the aim of eventually developing strategies to eliminate and/or prevent it.

## Close to the bone: the structure and composition of bone tissue

**Figure 1:**
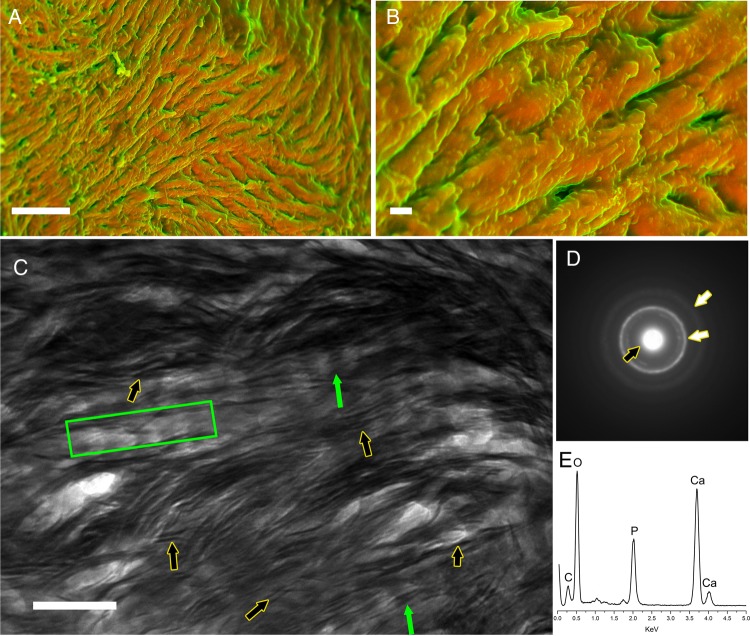
The nano-structure and composition of bone. (*A* and *B*) Representative density-dependent colour scanning electron micrographs of the surface of bone tissue from a human femoral head showing a homogenous density distribution of mineralized collagen fibres. The orange colour identifies the mineral, while green highlights the organic components of the tissue. Scale bar in (*A*): 10 µm and in (*B*) 1 µm. (*C*) Transmission electron microscopy image of bone. Green arrows and box indicate banded collagen fibres and black arrows highlight dark plate-like crystals. Notice how the dark crystals directly interact with the light collagen fibrils. Scale bar = 0.2 µm. (*D*) Selected area electron diffraction pattern of bone showing broad diffraction rings/halos (white arrows) indicative of disorder in the material's atomic structure. The central bright spot represents the source electron beam (black arrow). The broad diffraction rings/halos are created by reflections of disordered atoms within the material. (*E*) Energy dispersion X-ray spectroscopy elemental analysis of bone highlighting its composition: calcium, phosphorus, carbon, and oxygen.

Because of its inorganic nature and function as a structural material in the body, biologists and clinicians are not the only scientists who study bone. Quite uniquely, it has also received significant attention from the physical sciences communities. Chemists and engineers have long studied bone samples *ex vivo* using electron microscopy (nano-meter resolution imaging), energy dispersive X-ray spectroscopy (elemental analysis), and selected area electron diffraction (SAED, crystallographic structure) to precisely describe its hierarchical structure and composition (*Figure. 1*). As a result, the basic structure of bone, whether it be lamellar or woven, mature or immature, is well-established. In short, bone is composed of apatite crystals, which closely associate with collagen fibres^11^ (*Figure. 1A*–*C*), its elemental composition is predominantly calcium, phosphorus, carbon and oxygen (*Figure. 1E*), and its mineral is poorly crystalline (a measure of how regularly atoms align, *Figure. 1D*). Analyses of bones from different species show that bone has a similar structure, composition, and crystallinity across different sites in the body, and more widely, that it is comparable in nearly all vertebrates that have thus far been studied.^12^

## Heart of stone: the structure and composition of aortic valve calcific lesions

**Figure 2:**
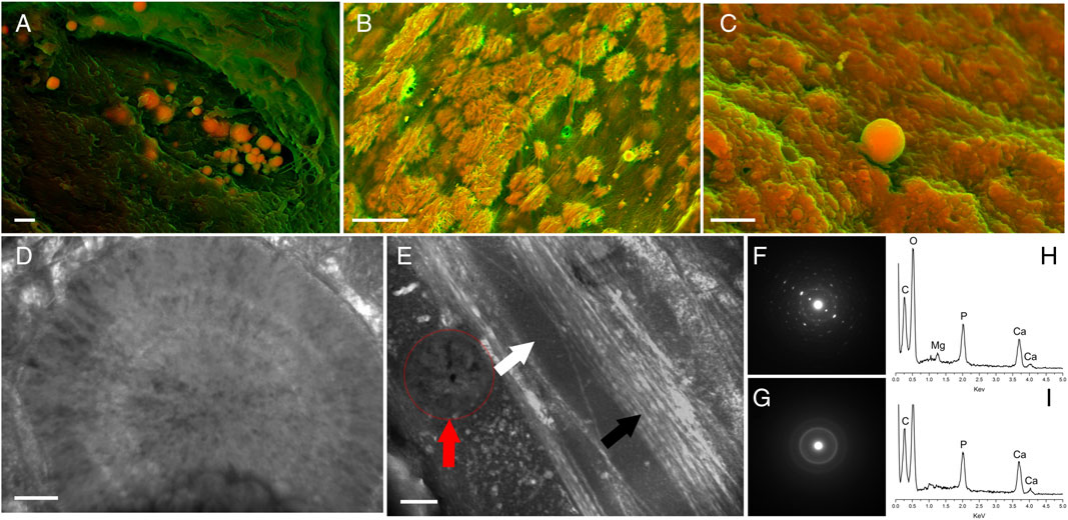
The nano-structure and composition of human aortic valve calcification. Representative density-dependent colour scanning electron micrographs of (*A*) calcified particles (arrow, scale bar = 1 µm), (*B*) calcified fibres (arrow, scale bar = 10 µm), and (*C*) compact calcification (white arrow) with a calcified particle (black arrow) in human aortic valve tissue (scale bar = 1 µm). Calcified areas appear orange, while the unmineralized extracellular matrix is shown in green. (*D*) Transmission electron microscopy image of a section through a calcified spherical particle. Notice that the particle is composed of a dense material all the way through. Scale bar = 0.2 µm. (*E*) Transmission electron microscopy image of compact calcification showing a trapped sphere (yellow arrow), compact amorphous calcium phosphate (white arrow), and collagen fibrils (black arrow). Notice how each component maintains its own structural identity. Scale bar = 0.2 µm. (*F*) Selected area electron diffraction of a calcified spherical particle. The diffraction pattern shows regularly spaced bright spots or *reflections* (white arrow) of the diffracted source electron beam (black arrow). Regularly spaced reflections are created by materials that have highly ordered atomic structure. (*G*) Selected area electron diffraction of compact calcification with a typical amorphous pattern, marked by broad diffraction rings/halos (white arrow) which reflect from the source electron beam (black arrow). Broad diffraction rings/halos are generated by the irregular arrangement of atoms within a material's structure. (*H*) Energy dispersion X-ray spectroscopy of a calcified particle showing that it is composed of calcium, phosphorus, and magnesium. (*I*) Energy dispersion X-ray spectroscopy of compact calcification presenting calcium and phosphorus.

As the structure and composition of bone has been well described, what then do the same techniques reveal about calcific lesions on the aortic valve? There are numerous electron micrographs of human calcific lesions available in the literature^13–20^ and we examined^14^ >30 recovered human aortic valves *ex vivo* using this non-quantitative technique. These images reveal that the inorganic component of calcific lesions takes one of three structures (*Figure. 2A*–*C*). The most common mineralized structure are spherical particles with sizes ranging from 100 nm to 5 µm (*Figure. 2A*). These ‘billiard ball-like’ particles have been observed in diseased samples, independent of disease severity. They are composed of calcium, phosphorus, oxygen, and magnesium (*Figure. 2H*), and their inner structure is formed from concentric layers of mineral distributed radially from the centre (*Figure 2D*).^14^ Although visible in SEM images published over the last 30 years,^16,21^ their presence was dismissed until recently, likely because standard TEM sample preparation fractures the spherical particles and histology techniques can miss them, as they are transparent like a quartz crystal.

‘Matrix vesicles’, membrane-bound spherical structures that contain clusters of needle-like mineral, play an important role in mediating bone formation. The dense, spherical mineral particles in aortic valve calcifications, however, fundamentally differ from matrix vesicles in composition and structure.^22–25^ Spherical particles tend to be larger than matrix vesicles, which are only ∼100 nm in diameter,^22,26^ and are solid completely through. In contrast, matrix vesicles mineralize along their membranes and often have hollow cores. Moreover, while matrix vesicles contain amorphous mineral,^22^ the atoms that comprise spherical particles are arranged in a highly ordered fashion, like a single crystal^14^ (*Figure 2F*). In fact, the spots (or *reflections*) in their SAED patterns demonstrate that their mineral is different from any other material found in the body. They are unquestionably far more crystalline than bone.

**Figure 3:**
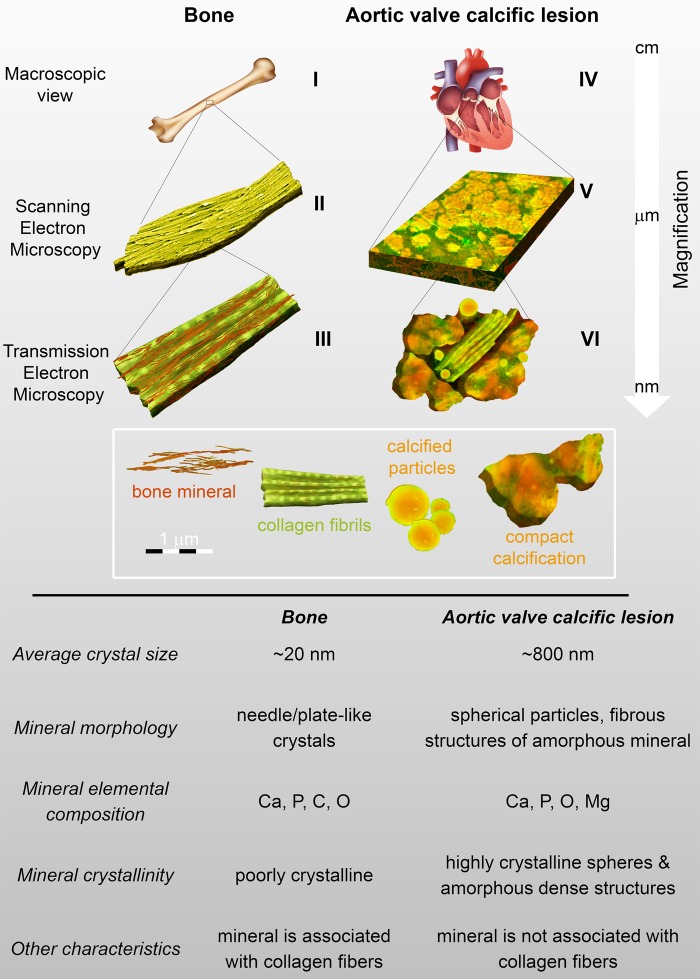
The hierarchical and ordered structure of bone compared with inhomogenous aortic valve calcification. Diagram shows the structure of each tissue at the macroscopic, micron (visible by scanning electron microscopy), and nano (visible by transmission electron microscopy) scales. I. Macroscopic schematic bone. II. Collagen fibrils covered by calcium phosphate mineral. III. Collagen fibrils associated with calcium phosphate mineral crystals at the nano-scale. IV. Macroscopic schematic of cardiac tissue. V. Micron-level structures observed in aortic valve calcific lesions. VI. Organization of calcific lesions which contain fibrous structures, calcified particles, and compact calcification. Table summarizes the characteristics of bone as compared with aortic valve calcific lesions.

The other two structures commonly found in calcific lesions are calcified fibres and compact calcification (*Figure 2B* and *C*).^14^ Most calcific lesions with these structures are likely formed by dystrophic mineralization, although 13% are reportedly true bone and possess features of mature lamellar bone, including mineral that templates on a collagen matrix.^7^ Most calcific lesions lack the nano-level organization typical of bone (*Figure 2E*), and although they are composed of calcium, phosphorus and oxygen, they do not contain detectable levels of magnesium (*Figure 2I*) and their mineral is poorly crystalline^14^ (*Figure 2G*). Interestingly, most mineralized structures in calcific lesions do not interact with collagen fibres. Instead, the mineral structures and organic components remain as isolated elements, each maintaining their own structural identity (*Figure 3*).

## A change of heart: do mineralized particles play a role in aortic valve calcification?

As conventional and emerging biological techniques are already providing exciting new insights into its pathogenesis, what additionally can we learn about aortic valve calcification from studying how bone forms? First, observations of ubiquitous spherical particles in diseased valves examined *ex vivo* suggest that calcific lesion formation does not follow a similar pattern to bone mineralization. One of the hallmarks of bone formation is that mineral crystals template on a collagen matrix.^11,27–28^ This is not the case in most calcific lesions in which the organic matrix does not associate with the mineral. Moreover, on the aortic valve, dense spherical particles are the first mineralized structure observed, and they are present even prior to the formation of calcific lesions.^14^ This observation offers the possibility that particles may play a role in mediating further mineralization. Indeed, their presence poses a number of questions: What is the role (if any) of these particles in calcification? Could they be initiators of calcification or a consequence?

Analyses of calcific lesions with physical science techniques also provide complementary evidence to that gained from traditional biological techniques. For example, it has confirmed histological studies that show that the composition and structure of most calcific lesions are not bone like. True bone formation reportedly only occurs in a small fraction of calcific lesions, and therefore may be a consequence of calcification rather than a cause.^29^ The biomaterials community has long known that implanting some forms of calcium phosphate intramuscularly can prompt bone formation.^30,31^ Moreover, the presence of hydroxyapatite nano-crystals has been shown to upregulate osteogenic gene expression in vascular smooth muscle cells.^32^ If a similar mechanism manifested in the vascular system, the localization of calcium phosphate to valves could trigger cell transdifferentiation, bone-specific protein formation and gene expression, and eventually actual bone formation. Although a mechanism by which this process might proceed remains unclear, interstitial valve cells have transdifferentiation and mineralization potential.^33^ Moreover, cell biologists have recently recognized that the stiffness of the substrate on which cells attach can affect their phenotype.^34,35^ Stiff surfaces that mimic the stiffness of the developing osteon, for example, direct mesenchymal stem cells to differentiate to osteoblasts. If the same were true in the aortic valve, stiff calcium phosphate might drive the transdifferentiation of local cells or prompt circulating cells to adhere and adopt an osteoblastic phenotype.

## The best of both worlds: the potential of an interdisciplinary approach

As vascular calcification appears to be more complex than either simple dystrophic mineral formation or a cell-mediated process of bone formation ‘in the wrong place’,^36^ how then can the field move forward to develop treatments for calcific diseases? The methods now available include biological, biochemical, materials, and clinical techniques. Combining methods and taking an interdisciplinary approach has already yielded promising new avenues of research, as detailed here, and their continued use could be similarly efficacious. For example, optical and fluorescence microscopy are powerful techniques for visualizing cells and identifying labelled proteins. However, they can only reveal insights at the micro-scale because of the inherent limitations of the wavelength of light. On the other hand, analytical electron microscopy is non-quantitative and often cannot provide biological information about cells and proteins, but it can produce ultra-high-resolution images of *ex vivo* samples and provide clear information about tissue morphology, mineral crystallinity, and elemental composition. Combining the two is possible and the techniques are not inaccessible to standard laboratories. Moreover, the two methods can be used sequentially on the same sample.^37^ To study aortic valve calcification, sections of recovered valves labelled for specific proteins could be correlated with electron micrographs highlighting the location, size and composition of calcified areas. Elemental analysis applied directly to cells and their labelled compartments might also identify if and how specific cellular organelles might contribute to mineral nucleation and/or subsequent propagation.^22^

Although an expectation that advances will come from applying physical science techniques to *ex vivo* aortic valve calcific lesions might seem like wishful thinking, methods borrowed from physics and chemistry are pervasive in clinical medicine. Ultrasound was first pioneered as a geological technique before it found a plethora of uses in medical imaging, and chemists utilized nuclear magnetic resonance (NMR) to verify the molecular structure of chemicals before the technique was applied in diagnostic radiology. Even electron microscopy techniques have been used for years to diagnose chronic renal allograft rejection and various viral infections. Indeed, in some circumstances electron microscopy remains the best diagnostic method available.^38–45^

## Conclusions

Interdisciplinary research approaches have revealed new insights into the pathogenesis of aortic valve calcification. In particular, they have led to the discovery that calcific lesions contain unique, highly crystalline spherical particles. Applying such techniques can complement existing approaches, providing additional insights into aortic valve calcification, and perhaps also pathways for clinical treatments. For example, although our understanding of spherical particles in the vascular system is limited, their highly crystalline nature suggests that they would be quite difficult to dissolve. A consequence of this might be that research efforts that aim to prevent calcification or limit its progression, rather than trying to remove them, may be more clinically effective. Such new ways of looking at long-standing research problems may foster advances in cardiovascular and biomedical research.

## Authors' contributions

E.G., S.B. handled funding and supervision; E.G., S.B. conceived and designed the research; E.G., S.B. drafted the manuscript; E.G., S.B. made critical revision of the manuscript for key intellectual content.

## 

### Funding

Funding to pay the Open Access publication charges for this article was provided by the Wellcome Trust.

**Conflict of interest:** none declared.
